# Shared detection of *Porphyromonas gingivalis* in cohabiting family members: a systematic review and meta-analysis

**DOI:** 10.1080/20002297.2019.1687398

**Published:** 2019-11-07

**Authors:** Maha Bennani, Hélène Rangé, Vincent Meuric, Francis Mora, Philippe Bouchard, Maria Clotilde Carra

**Affiliations:** a Department of Periodontology, Service of Odontology, Rothschild Hospital, Paris, France; b U.F.R. of Odontology, Université de Paris, Paris, France; c EA 2496 Laboratory Orofacial Pathologies, Imaging and Biotherapies, Faculty of Dental Surgery, Paris Descartes University, Montrouge, France; d Microbiology UPRES-EA 1254, Université Européenne de Bretagne, Université of Rennes 1, Rennes, France; e Inserm, Population-based Epidemiologic Cohorts Unit, Villejuif, France

**Keywords:** Periodontitis, periodontal pathogens, *Porphyromonas gingivalis*, family members, proband, spouses, microbiota

## Abstract

**Introduction**: Periodontitis is an inflammatory dysbiotic disease. Among putative dysbiosis causes, transmission of *Porphyromonas gingivalis* between individuals of the same family remains unclear. The aim of this systematic review and meta-analysis is to assess the likelihood of shared detection of *Porphyromonas gingivalis* among cohabiting family members.

**Methods**: A literature search was conducted on different databases up to September 2018. Articles assessing the presence of *P.gingivalis* between members of the same family were screened. Only English literature was retrieved, whereas no limits were applied for bacterial sampling and detection methods.

**Results**: Overall, 26 articles published between 1993 and 2017 met the inclusion criteria. Of these, 18 articles were used for meta-analyses. Based on bacterial culture, the likelihood of an intra-familial transmission of *P.gingivalis* once a member of the family harbors the bacterium is estimated at 63.5% (n = 132 pairs of family members); this drops to 45% when pooling together culture and Polymerase-Chain-Reaction (n = 481 pairs), whereas it is estimated at 35.7% when genotyping is applied (n = 137 pairs).

**Conclusion**: Pooled results suggest that the likelihood of detecting *P.gingivalis* within within family members is moderately frequent. Personalized periodontal screening and prevention may consider intra-familial co-occurrence of *P.gingivalis* as feasible.

## Introduction

Periodontitis is a multifactorial inflammatory disease associated with dysbiotic subgingival microbiota and characterized by progressive destruction of the tooth-supporting apparatus [1]. The transition from periodontal health to periodontal disease is associated with the shift from a symbiotic microbial community, mostly composed of facultative anaerobic bacterial genera like *Actinomyces* and *Streptococci*, to a dysbiotic microbial community composed of anaerobic genera from the phyla *Firmicutes, Proteobacteria, Spirochaetes, Bacteroidetes* and *Synergistetes* [2,3]. This shift is likely induced by pathogenic bacteria able to trigger quantitative and qualitative alterations in the commensal communities and consequently initiate the destructive inflammatory processes at the level of the periodontium. The most studied ‘keystone pathogen’ for periodontitis is *Porphyromonas gingivalis (P. gingivalis*), a highly virulent Gram-negative asaccharolytic bacterium [4,5].

Thus, colonization of the mouth by putative pathogens may lead to periodontal disease in susceptible recipients. The infectious etiology of periodontitis is consistent with the familial clustering of the disease, although the classical meaning of infection transmission cannot be applied in periodontitis given the complexity of the multifactorial aspect of oral microbiome and host interactions [6,7]. However, the possibility of an inter-individual transfer of oral microbiota remains relevant and investigating how and how often the main periodontal pathogens can be transmitted may provide further information to assess the patient’s risk profile.

The present systematic review and meta-analysis aims to answer the following question: what is the likelihood of shared detection of *P. gingivalis* among cohabiting family members?

## Materials and methods

### Study design

This is a systematic review of studies focusing on the intra-familial co-occurring detection of *P. gingivalis* between spouses, parents-infants, and among siblings. The PRISMA statement checklist was followed in the reporting of this systematic review.

### Eligibility criteria for study inclusion

Clinical trial, longitudinal studies (retrospective or prospective), cross-sectional studies, case–control studies, case-series (reporting about at least four families cases) were eligible for inclusion. Review articles, case report, and studies treating of a possible intrauterine transmission of periodontal pathogens or transmission between humans and animals were not considered. Studies focusing on periodontal bacteria other than *Porphyromonas gingivalis* were also excluded.

The eligibility criteria by applying the PICO framework were the following:

Population: Family members living together with at least one member carrying *P. gingivalis* (so-called proband), with or without periodontitis.
Intervention: All microbiological detection methods were considered (e.g. culture, polymerase chain reaction (PCR), serotyping, genotyping, ribotyping).
Comparator: Not applicable
Outcomes: Likelihood of intra-familial detection of *P. gingivalis.*

In the majority of the studies, the co-occurring detection of *P. gingivalis* between two family members (concordance rate) was used to support the hypothesis of a direct intra-familial transfer, namely horizontal transmission when occurring between spouses and vertical transmission when occurring between parent and infant. In the present study, we avoid to talk about bacterial transmission, we rather estimate the likelihood of a simultaneous detection of the bacterium in the proband (carrier of *Porphyromonas gingivalis*) and his/her family relative.

### Information sources

The literature search for the present systematic review was conducted on the following online available databases: MEDLINE (through PubMed), EMBASE, Cochrane Oral Health Group Specialized Register, ProQuest Dissertations and Thesis Database. A grey literature search was also performed by searching the OpenGrey database. Studies meeting the selection criteria were reviewed if written in English. The study protocol was begun in November 2017; literature search was performed on September 2018. The systematic review protocol was registered in Prospero on 17 April 2018 (registration number: ID = CRD42018092737).

### Search strategy

A specific research equation was formulated for each different database, using the following keywords and/or MeSH terms: transmission, aggregation, *Porphyromonas gingivalis*, family/families, spouse, periodontitis, oral bacterial colonization, oral bacteria, microbiome. In addition, the reference lists of eligible studies and relevant review articles (not included in the systematic review) were crosschecked to identify other relevant studies.

### Study selection and quality assessment

Studies were selected by two independent reviewers (M.B. and M.C.C.). At first, the titles and abstracts of the retrieved studies were independently and blindly screened for relevance. To enhance sensitivity, records were removed only if both reviewers excluded them at the title/abstract level. Subsequently, both reviewers performed a full-text analysis of the selected articles. Disagreements about inclusion or exclusion of a study were resolved by consensus. The two reviewers independently assessed the risk of bias, using appropriate tools according to the study design. Most of the study were observational studies, thus the quality assessment was carried out by using the star template of the Newcastle-Ottawa Scale (NOS) tool. The NOS scores of 1 to 3, 4 to 6, and 7 to 9 were judged for low, moderate, and high quality of studies, respectively.

### Data extraction and analysis

Data from the selected studies were processed for quality synthesis. Relevant findings and outcomes were extracted from the original studies and summarized in tables. Extracted data included first author, year of publication, patient numbers, study design, periodontal status of the probands, bacteria sampling, detection methods, transmission rate. Whenever the transmission rate was not provided or not estimable from the article data, corresponding authors were contacted by email to obtain data on the proportion of family members sharing *P. gingivalis*. Consequently, whenever possible, studies were grouped by detection method and type of familial relationship, and a proportion meta-analysis was run. The meta-analysis and forest plots were derived by using MedCalc software (version 17.9 for Windows). The pooled proportion of likelihood of co-occurrence of *P. gingivalis* was analyzed with the estimation of 95% confidence interval (CI). Random effects model (DerSimonian and Laird method) [8] was used. Heterogeneity of the studies was tested by Cochran’s *Q* statistic, and *I*^2^. Sensitivity analysis was also performed by sequentially excluding studies that may be responsible for heterogeneity. Funnel plots were used to examining the presence of publication bias.

## Results

### Article search and selection

**Figure 1.**
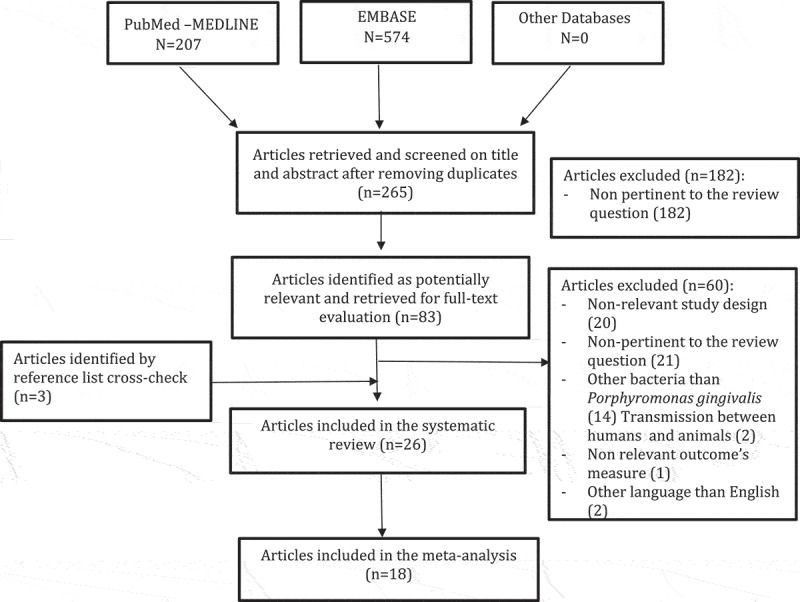
Flowchart of the literature search and article selection

Overall, 265 articles were initially identified; of these, 182 were rejected upon titles and abstract because not relevant for the review topic. The remaining 83 articles were screened at the full-text level; 26 were selected for the systematic review. Of these, 18 articles were used for pooled data analyses. Overall, the selected articles were published between 1993 and 2017. The flow chart of the study selection process is shown in Figure 1.

### Study characteristics

All the selected studies were observational studies, including 7 cross-sectional, 10 case series, 5 case-control, and 4 cohort studies. Co-occurrence of *P. gingivalis* between spouses was explored in 18 studies, including 334 couples; co-occurrence between siblings was explored in 3 studies. Detection concordance was explored between parent and infant(s) in 14 studies, including 625 pairs of parents-children. Details of study characteristics according to the type of familial relationship are displayed in Tables 1–3.

**Table 1.** Summary of the included studies assessing the simultaneous detection of *Porphyromonas gingivalis (Pg)* between adult couples of the same family

Author and year	Study design	Number of couples	Periodontal status of the proband	Periodontal status of the spouses	Bacteria sampling	Detection methods	Proportion of intra-familial transmission
Petit et al. 1993	Case series	4	Adult periodontitis	Adult periodontitis	4 periodontal pockets Mucous membrane Dorsum of the tongue	Culture DNA-REA	Culture: 3/4 DNA-REA: 0/4
Van Steenbergen et al. 1993a	Case series	18	Untreated adult severe periodontitis (at least 10 pockets of ≥5 mm and at least one pocket with ≥ 4 mm of attachment loss and sub-gingival presence of *Pg)*	5/18 spouses diagnosed as periodontitis patients	4 deepest pockets (by paper points) Buccal mucosal Dorsum of the tongue Tonsillar area Saliva	Culture DNA-REA	Culture: 8/18 DNA-REA: 6/18
Van Steenbergen et al. 1993	Case series	8	Severe adult periodontitis and subgingival presence of *Pg*		4 deepest pockets (by paper points) Buccal mucosa Dorsum of the tongue Tonsillar area Saliva	DNA-REA AP-PCR Ribotyping	DNA-REA: 6/8 AP-PCR: 6/8 Ribotyping: 6/8
Saarela et al. 1993	Case series	4	Advanced periodontitis and subgingival presence of *Pg*	Advanced periodontitis and subgingival presence of *Pg*	Deepest, the most inflamed periodontal pockets (by curette) Stimulated saliva	Serotyping Ribotyping	Ribotyping: 2/4 (married couples for at least 10 years)
Von Troil Lindén et al. 1995	Case-control	10 vs. 10	Advanced periodontitis probands vs. periodontally healty controls	Not specified	6 deepest and most inflamed periodontal pockets (by sterile curette) Stimulated saliva	Culture	Probands: 6/10 on subgingival samples 2/10 on salivary samples Controls: 1/10 on subgingival samples 0/10 on salivary samples
Asikainen et al. 1996	Case series	15	Adult periodontitis (AAP Classification 1989)	Adult periodontitis (85% of spouses)	4 to 6 deepest periodontal pockets (by curette) Dorsum of the tongue From healthy subjects: mesial subgingival sites of the first molars.	Culture AP-PCR	Culture: 9/15 AP-PCR: 2/15
Van der Velden et al. 1996	Longitudinal study Time frame: 1987–1994	22	No specific definition (based on clinical parameters: plaque index, calculus, probing depth, bleeding on probing, loss of attachment)	Not specified	4 periodontal pockets (paper points)	Culture	Culture: 9/22
Tuite-McDonnell et al. 1997	Cross-sectional	43	17% of individuals had at least one site with an attachment level or PPD ≥5.5 mm	Not specified	all teeth (paper point)	PCR	PCR: 32/43 Spouses were significantly more frequently colonized by *Pg* (RR: 4 [95%CI: 2.3–7]) than what would be expected if *Pg* was randomly distributed in the study population. No relationship was observed between the length of time a couple had been married and their concordance of colonization.
Von Troil Linden et al. 1997	Longitudinal experimental study Six month follow up study	8	Advanced periodontitis	3 spouses: moderate periodontitis undergone a mechanical treatment combined with metronidazole. 7 spouses: mild to moderate periodontitis were untreated	6 deepest and most inflamed pockets Paraffin-stimulated saliva	Culture	Culture: 4/8
Van Winkelhoff et al. 1999	Longitudinal study. Time frame: 1987 – 1994	19	No selection on the periodontal status	Not specified	4 sub-gingival samples using 1 paper point per pocket	Culture Serotyping	Culture: 15/19 Serotyping: 0/19
Asano et al. 2003	Cross-sectional	25	Periodontitis defined as at least 4 pockets with PPD > 4 mm and radiographs showing the presence of at least one intrabony defect > 4 mm.	Not specified	3 deepest pockets (by paper points), or gingival sulci	Culture PFGE FimA genotyping	Culture: 14/25 PFGE: 6/14 The frequency of FimA type II in isolates from the 6 couples with identical PFGE patterns was 83.3%, which was higher than for the periodontitis unrelated patients (46.8%) (positive for *Pg*).
Park et al. 2004	Case series	16	Chronic and aggressive periodontitis	Not specified	3 sterile paper points into the mesial sulcus of diseased sites of the probands and the deepest sites of their family members	PCR (Isi 1126 specific primer)	PCR (Isi 1126): 5/16
Rijnsburgurger et al. 2007	Case series	6	Untreated severe periodontitis	Not specified	Subgingival samples Dorsum of the tongue Buccal mucosa, Tonsils Saliva	REA AFLP-PCR	REA: 4/6 AFLP-PCR: 4/6 (showing a similarity >85%)
Van Winkelhoff 2007	Cross-sectional	13	Population without regular dental care	Not specified	Deepest bleeding sites with the greatest amount of attachment loss	AFLP-PCR	AFLP-PCR: 0/13
Van Winkelhof 2008	Longitudinal study. Time frame: 1994–2002	27	Population without regular dental care	Not specified	Deepest bleeding sites with the greatest amount of attachment loss	Culture AFLP-PCR	Culture: 1994: 13/27 2002: 18/27 AFLP-PCR: 1994: 0/27 2002: 3/27
Martelli et al. 2012	Case series	9	Untreated periodontitis	Untreated periodontitis	One pocket per quadrant (by paper point)	PCR FimA genotyping	PCR: 6/9 FimA type II: 6/9
Feng et al. 2015	Case series	10	Aggressive periodontitis	Not specified	Subgingival samples Saliva	PCR FimA genotyping	PCR: 7/10 FimA genotyping: 7/10

AP-PCR stands for arbitrarily primed polymerase chain reaction; BOP for bleeding on probing; PPD for probing pocket depth; PI for plaque index; *Pg* for *Porphyromonas gingivalis. Aa* for *Agregatibacter actinomycetemcomitans. Pi* for *Prevotella intermedia*. AFLP: Amplified fragment length polymorphism, DNA REA: Deoxyribo Nucleic Acid Restriction Endonuclease Analysis; Fim A: Fimbriae A.

**Table 2.** Summary of the included studies assessing the simultaneous detection of *Porphyromonas gingivalis (Pg)* between parents and children of the same family

Author and year	Study design	Number of children	Periodontal status of the parents	Periodontal status of the children	Bacteria sampling	Detection methods	Main findings (Number of positive/Total)
Petit et al. 1993	Case series	10	Adult periodontitis	Severe generalized juvenile periodontitis (GJP) with presence of *Aa* and *Pg*	4 periodontal pockets Mucous membrane Dorsum of the tongue First molar if no PPD	Culture DNA-REA	Culture: 7/10 DNA REA: 5/10
Petit et al. 1994	Case series	36	Untreated adult severe periodontitis (at least 10 pockets of ≥5 mm and at least one pocket with ≥ 4 mm of attachment loss and subgingival presence of *Pg* or *Aa* or *Pi)*	Not specified	4 periodontal pockets Mucous membrane Dorsum of the tongue Tonsilla area Saliva First molar if no PPD	Culture	Culture: 1/36
Asikainen et al. 1996	Case series	19	Adult periodontitis (AAP Classification 1989)	5% of children exhibited periodontal destruction	4 to 6 deepest periodontal pockets (by curette) Dorsum of the tongue First molar if no PPD	Culture AP PCR	Culture: 1/19 No AP PCR (not enough samples)
Tuite-McDonnell et al. 1997	Cross sectional	103 oldest children with their mothers 101 oldest children with their fathers 98 adult children with their mothers 69 adult children with their fathers	17% of individuals had ≥ 1 site with an attachment level or PPD ≥ 5.5 mm	Not specified	All teeth (Paper points)	PCR	PCR (Oldest child with the mother): 20/103 PCR (Oldest child with the father): 17/101 PCR (Adult child with the mother): 25/98 PCR (Adult child with the father): 14/69
Umeda et al. 2004	Cross sectional	56	Only 4 parents had PPD > 4 mm	Not specified	Saliva + supragingival plaque (Children) Saliva (Parents)	PCR	PCR (Parent): 54.8% PCR (Children): 7.1%
Okada et al. 2004	Cross sectional	28	1 healthy individual 4 gingivitis 7 periodotnitis	6 healthy individuals 6 gingivitis 6 periodontitis	Supra-gingival plaque collected with sterile tooth brushes (1 min)	PCR	PCR: 6/28 (21.4%)
Park et al. 2004	Case series	14	Chronic and aggressive periodontitis	Not specified	3 Sub-gingival samples (Paper points)	PCR with Isi 1126 specific primer	PCR Isi 1126: 11/14
Tamura et al. 2006	Cross sectional	106	Not specified. All patients were recruited from the Periodontics Clinic of Osaka University Hospital.	No children had periodontitis	Saliva samples	PCR	PCR (Mothers): 42.5% PCR (Children): 2.7%
Kobayachi et al. 2008	Cross sectional	78	Not specified	No children had moderate or severe gingivitis	Sub-gingival plaque (first molar for children; first and second molars for mothers)	PCR	PCR (Mothers):17.6% PCR (Children): 9%
Belcheva et al. 2012	Case control	11	Group of diseased parents and their children Group of healthy parents and their children	Not specified	Deepest pockets (First molars and lower incisors) Sulci between premolars and molars when healthy	Culture PCR	Culture (Children): No detection PCR (Children): 2/11 The study did not find statistically significant difference (*p*> 0.05) in the prevalence of *Pg* between children with healthy parents and children whose parents presenting with periodontal disease
Monteiro et al. 2014	Case control	15	Generalized aggressive periodontitis (GAP) vs healthy	Not specified	Stimulated saliva (without dental hygiene-no brushing 2 hours before sampling)	PCR	PCR (Periodontitis parent): 50% PCR (Infant): 30% Parents with GAP showed statistically higher *Pg* salivary concentration than periodontally healthy parents (*p* < 0.05). Children of GAP families, however, exhibited similar *Pg* concentration than healthy children (*p* > 0.05).
Monteiro et al. 2015	Case control	15	Generalized aggressive periodontitis vs healthy	Not specified	Stimulated saliva (without dental hygiene-no brushing 2 hours before sampling)	PCR	PCR (Periodontitis parent): 80% PCR (Infant): 60%
Feng et al. 2015	Case series	10	Aggressive periodontitis	Not specified	Saliva Sub-gingival samples (First molars)	PCR with Fim A genotype Specific primers	PCR (Fim A genotyping): 7/10
Al Yahyoufi. 2017	Case series	11	Chronic and Aggressive Periodontitis	Not specified	4 Sub-gingival samples (Paper point in each quadrant)	DNA probe	DNA probe: 7/11

AP-PCR stands for arbitrarily primed polymerase chain reaction; *Pg* for *Porphyromonas gingivalis. Aa* for *Aggregatibacter actinomycetemcomitans. Pi* for *Prevotella intermedia*. AFLP: Amplified fragment length polymorphism; Fim A for Fimbriae A; DNA REA for Deoxyribo Nucleic Acid Restriction Endonuclease Analysis.

**Table 3.** Summary of the included studies assessing the simultaneous detection of *Porphyromonas gingivalis (Pg)* between siblings

Author and Year	Study Design	Number of Subjects	Periodontal Status of the Probands	Periodontal Status of the Siblings	Bacteria Sampling	Detection Method	Main Findings
Van winkelhoff et al. 1999	Longitudinal study 1987–1994	29 sibships	No selection on the periodontal status	Not specified	4 sub-gingival samples using 1 paper point per pocket	Culture Serotyping	Culture: 26/29 Serotyping: 3/29
Van Winkelhoff. 2007	Cross sectional study	10 sibships 30 siblings	Population without regular dental care	Not specified	Deepest bleeding site with the greatest amount of attachment loss	AFLP typing	2/10 Sibships 13/30 Siblings
Van Winkelhoff. 2008	Longitudinal study 1994–2002	23 sibships 54 siblings	Population without regular dental care	Not specified	Deepest bleeding site with the greatest amount of attachment loss	Culture AFLPT typing	Culture (1994): 22/23 Culture (2002): 22/23 AFLP PCR (1994): 6/23 AFLP PCR (2002): 6/23

*Pg* stands for *Porphyromonas gingivalis*. AFLP for Amplified fragment length polymorphism.

Regarding the clinical periodontal status of the *proband*, the classical periodontal parameters, i.e. periodontal pocket depth, clinical attachment level, gingival inflammation, and bleeding were recorded in some studies. In few of them, radiographic parameters were also considered. Classification of periodontitis according to the AAP Classification 1999 defining chronic or aggressive periodontitis was used in three studies [9–12]; adult periodontitis was described in four studies, whereas advanced periodontitis in three. Several studies did not provide a clear definition of the periodontal disease used.

Bacterial sampling was carried out on supra-gingival and/or subgingival plaque, stimulated saliva, or sampled from dorsum of the tongue, buccal mucosa, or tonsillar area. Detection methods included culture in 10 studies (38.4%), and Polymerase Chain Reaction (PCR) in 11 studies (42.3%). DNA restriction enzyme analysis (REA-DNA) was used in 4 studies (15.3%), pulsed field gel electrophoresis (PFGE) in 1 study, arbitrarily primed polymerase chain reaction (AP-PCR) in 2 (7.6%) studies, amplified fragment length polymorphism (AFLP-PCR) in 3, strain-specific identification of *P. gingivalis I* Isi 1126 PCR in 1, and Fim A genotyping in 3 studies. Serotyping characterization and ribotyping were reported in 2 studies, respectively.

### Quantitative analyses

Forest plots were built pooling together intra-familial data according to the detection method used. Concerning the co-occurring detection between adult partners or spouses living together, the meta-analysis showed that the likelihood of detecting *P. gingivalis* in the partner/spouse when a proband was harboring *P. gingivalis* was 53.58% (95%CI: 44.98%-62.95%; I^2^: 0%; n = 99 pairs of partner/spouse) by culture, and 58.88% (95%CI: 48.02%-69.32%; I^2^: 39.6%; n = 142 pairs) when considering culture + PCR.

By applying genotyping, the likelihood of detecting the same strain of *P. gingivalis* between the members of the couple dropped at 29.02% (95%CI: 17.68%-41.87%; I^2^: 43.2%; n = 91 pairs). Using Fim A genotyping method, the same Fim A was retrieved in 57.15% (95%CI: 40.87%-72.68%; I^2^: 0.2%; n = 33 pairs) of the cases. Overall, the same ribotype of *P. gingivalis* was found in 64.7% (95%IC: 38.83%-86.63%; I^2^: 0%; n = 12 pairs) of couples when both adults were positive for *P. gingivalis*.

Concerning the parent-infant co-occurrence, when at least one parent was colonized by *P. gingivalis*, the likelihood that also one child harbored *P. gingivalis* was estimated at 20.15% when considering three studies using culture (95%CI: 0.31%-58.89%; I^2^: 90.4%, n = 65 pairs of parent-infant) [13–15], and 5% when considering two studies (95%CI: 0.91%-12.11%; I^2^: 0%; sensitivity analysis on 55 pairs) [13,15]. The detection of *P. gingivalis* using PCR amplification showed a likelihood of 22.46% (95%CI: 17.46%-27.90%; I^2^: 0%, n = 240 pairs), whereas when combining both detection methods (culture + PCR), the estimated likelihood was 24.05% (95%CI: 13.41%-36.64%; I^2^: 80.5%, n = 316 pairs) [13–19]. Based on two studies only, the same *P. gingivalis* genotype of the parents was shared in 64.95% of children harboring *P. gingivalis* (95%CI: 37.73–87.74%; I^2^: 50.5%; n = 24 pairs) [12,14].

**Figure 2.**
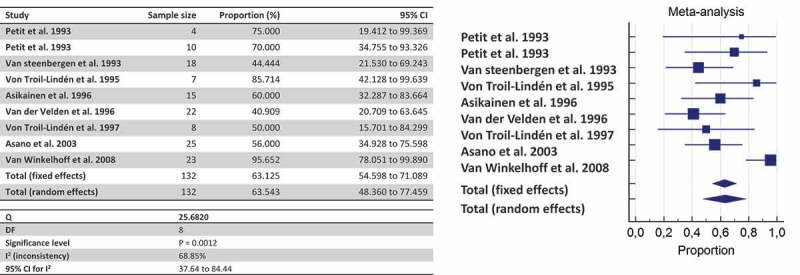
The likelihood of co-occurrence of *P. gingivalis* once a family member harbors *P. gingivalis* assessed by culture

**Figure 3.**
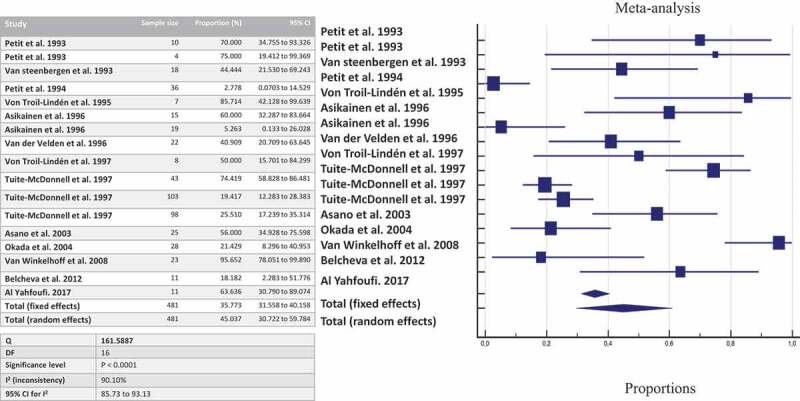
The likelihood of co-occurrence of *P. gingivalis* once a family member harbors *P. gingivalis* assessed by culture and PCR

**Figure 4.**
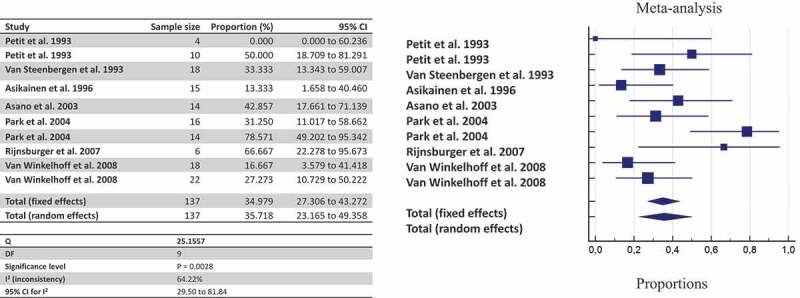
The likelihood of co-occurrence of *P. gingivalis* once a family member harbors *P. gingivalis* assessed by genotyping

By pooling together all data about shared detection both between-spouses and parent-infant, the likelihood of detecting *P. gingivalis* once a family member harbors the bacterium is estimated at 63.54% by culture (Figure 2), 45.03% by culture + PCR (Figure 3), and 35.71% by genotyping (Figure 4).

### Study quality assessment

Two reviewers (M.B. and M.C.C.) scored the methodological qualities of the included studies. The NOS score varies from 3 to 5, and over 9; thus, the studies were qualified as low (n = 17) and moderate (n = 9) quality studies. Detailed information regarding the quality assessment of the included studies is reported in Supplement Table 1.

## Discussion

The present systematic review and meta-analysis focuses on the likelihood of detecting one of the major periodontal pathogens, namely *P. gingivalis*, among cohabiting family members, and demonstrates that the co-occurrence of *P. gingivalis* among couples, children, or siblings ranges between 42% and 62% when a family member of is carrying *P. gingivalis* (the so-called proband). However, only in 35% of cases, these members are sharing the same genotype of the bacterium.

These data quantify the likelihood of co-occurring detection and support the hypothesis that an intra-familial bacterial transfer may occur. However, this appears to be highly variable both between spouses or couples and between parents and children despite their intimate cohabitation for long periods of time. The observed variability could be explained in several ways. First of all, bacterial transfer and detection does not necessarily mean persistent colonization up to the detectable levels of the pathogens but could be caused by repeated inoculation [6]. Indeed, bacterial transmission results from a combination of a sufficiently large and concentrated inoculum enabling bacterial survival during colonization, with a favorable oral environment of the recipient, which is dependent on the resident microbiota and the host defenses and characteristics [6,20]. Moreover, several behavioral and environmental patterns may influence the likelihood of bacterial colonization, such as hygiene habits, proximity, and intimacy differences among family members. However, as observed for other body sites, like skin or fecal microbiota, the family unit have a strong effect on human microbial community composition: indeed, family membership may explain a large proportion of the variability in bacterial diversity, with family members tending to harbor similar microbiota [7,21,22]. Indeed, the chance of sharing the same bacterial genotype appears to be greater among related individuals than unrelated ones or if *P. gingivalis* was randomly distributed in the population [13,19,23].

### Sampling and detection of porphyromonas gingivalis

Although the possible ways for transferring of *P. gingivalis* remains unclear, the role of saliva as a vehicle of bacterial spread is probable and it is supported by the fact that *P. gingivalis* can be cultured from salivary samples, indicating that this bacterium survives in the saliva during transportation to a new host [24]. Indeed, the probability of inoculation appears to be directly related to the salivary bacterial load, with a greater risk of colonization in the recipient for greater bacterial loads [20,24]. Then, *P. gingivalis* is able to spread intra-orally and colonize supra-gingival and sub-gingival plaque at sites with and without periodontal attachment loss, although, it is more likely to find *P. gingivalis* in deep pockets rather than shallow ones [25]. Consequently, the eventual eradication (i.e., a bacterial load under the detection level) of this pathogen by efficacious periodontal treatments may prevent its spread among individuals [6,24].

It must be noted that the detection of periodontal pathogens is drastically influenced by the methods applied to sample and recover them (e.g., culture, PCR, DNA probe checkerboard) [26]. As deemed from Tables 1 and 2, the sampling and detection methods applied in the studies included in the present systematic review are highly heterogeneous. This implies to analyze data by sub-groups of comparable detection methods while avoiding global comparisons. However, no distinction could be made according to the bacterial sampling site (e.g. saliva, subgingival plaque) because almost all studies provided pooled results of all samples examined (although multiple sites were sampled in most cases). Moreover, we must highlight that the included studies were conducted in a time span of 24 years during which microbiological techniques have drastically evolved as well as our knowledge about the role of specific periodontal pathogens and the complexity of the oral microbiota [27].

Several studies used culture to detect and quantify *P. gingivalis*, but no distinction between clones was made. The sensitivity of bacterial culturing is rather low, with detection limits averaging at 10^3^–10^4^ bacterial cells [26,28]. On the contrary, methods based on immune diagnosis (serotyping) and molecular analysis, such as PCR and ribotyping are highly sensitive and specific [26]. No study to date investigated the concordance of bacterial colonization and the likelihood of sharing the same periodontal microbiota between family members by applying modern methods, such as high throughput sequencing methods.

### Clinical implications of familial porphyromonas gingivalis sharing

If an intra-familial transmission of *P. gingivalis* is possible and probable, the available studies present a design and an overall level of quality that do not allow to conclude about ta specific intra-familial transmission pattern. Nevertheless, they provide evidence about the likelihood of a shared detection of *P. gingivalis* among family members.

A key question is whether the sharing of *P. gingivalis* can affect the periodontal health of the individuals becoming colonized. Some studies found that spouses of patients with advanced periodontitis have a worse periodontal status than spouses of periodontal healthy individuals [29]. Others did not demonstrate that the periodontal condition of the spouse was influenced by that of the partner [15,19]. When the relationship between the duration of marriage and the chance of transmission was explored, the study of Tuite-Mcdonnel et al. found no relationship between the frequency of co-occurring detection and the length of marriage, suggesting that cross-colonization likely occurred in the early years of marriage and remains stable over time [19].

When the frequency of presence of *P. gingivalis* in spouses of colonized proband was compared to unrelated patients, the spouses were significantly more frequently colonized by *P. gingivalis* than what would be expected if *P. gingivalis* was randomly distributed in the population [19,23]; but this was not observed in all studies [13].

Indeed, transfer of *P. gingivalis* from an individual to another does not necessarily translate into stable colonization and periodontal breakdown. It may persist an equilibrium between the host and the resident microbiota despite the repeated inoculation of *P. gingivalis*, especially in periodontally healthy individuals. However, we may consider *P. gingivalis* colonization as a potential risk factor, as it is known that this bacterium is causal in periodontitis initiation, progression, recurrence, as well as in peri-implantitis [5,30].

*P. gingivalis* can be possibly shared between parents and children. This raises another important question: does an early inoculation increase the chances of a permanent colonization and development of periodontitis later on in children? Early studies using culture probably underestimated the prevalence of *P. gingivalis* in young subjects, seldom detecting it before puberty [31,32]. Later on, studies relying upon more sensitive techniques, such as DNA-based technologies, demonstrated the presence of *P. gingivalis* in a large fraction of young subjects and showed it to be equally common in children of all ages [19]. Indeed, *P. gingivalis* can be detected in children aged of 20 days as of 18 years [33–35]. An epidemiological study, using DNA probe checkerboard assay, found that 71% of the 18- to 48-month-old children were infected with at least one periodontal pathogen [36]. *P. gingivalis* detection rates was estimated at 68.8% in children. A study evaluating the influence of mother’s periodontal clinical status on the prevalence of periodontal pathogens in newborns (aged of 3 months) showed that *P. gingivalis* was the most prevalent pathogen followed by others periodontal bacteria (*Prevotella intermedia, Tannerella forshythia, Campylobacter rectus*, *Aggregatibacter actinomycetemcomitans)* [37]. They concluded that the maternal clinical periodontal status is a significant indicator of the oral microbiota composition in the newborn children.

If the colonization in young children occurs, it appears to be composed by the same *P. gingivalis* strain of the parent(s) only transitory, whereas it becomes more stable during teenage years, possibly as deeper pockets develop [33]. Indeed, the presence of *P. gingivalis* is favored by the presence of deep probing depths, and thus it is likely that transmission from a proband infected individual to a periodontally healthy one is possible in terms of transient presence in the oral cavities, but in the absence of a permanent niche, such as a deep pocket, it may no longer survive.

It is noteworthy that the exposure and chance of colonization of *P. gingivalis* may vary in relation to the proband status, the severity of the periodontal disease, and the administration of a successful periodontal treatment. Moreover, bacterium-specific virulence factors may facilitate the familial sharing of *P. gingivalis*. Particularly, the fimbriae A (FimA), a specific component of the cell surface [38], seems to play a strategic role in the colonization and invasion of the periodontal tissues [39,40]. The FimA gene has been classified into six types (I to V and Ib) [41]. A higher rate of type II FimA was detected in couples who shared the same strains of *P. gingivalis* [9,23,42], being this strain mainly associated with severe periodontitis [43]. Conversely, the type IV FimA was not found in couples with identical PFGE patterns [23].

The present systematic review has some limitations. Retrieved data are heterogeneous: different methods for bacterial sampling, detection, and quantification were applied, requiring subgroup analyses. Findings are based upon a low-to-moderate level of evidence coming mainly from retrospective, cross-sectional studies or case series with a small sample size, which hamper any conclusion on the specific colonization patterns or on the eventual deterioration of periodontal health after the occurrence of *P. gingivalis* sharing within the family. Moreover, it should be stressed that a shared detection of pathogens (or colonization concordance) in cohabiting family members does not necessarily prove the transmission of these pathogens. Finally, the majority of the available studies were published in the ‘90. Due to relevant technical progress in microbiology in the last decade, the presented findings should be replicated and updated in lights of more recent knowledge about the diversity and richness of the oral microbiota [44].

## Conclusion

The present systematic review and meta-analysis supports the co-occurring detection of *P. gingivalis* within family members. Due to the role of *P. gingivalis* in the etiology of periodontitis, the likelihood of sharing *P. gingivalis* should be considered in the assessment of patient’s periodontal risk.

## Supplementary Material

Supplemental Material
