# Supplementary material for: Shared detection of Porphyromonas gingivalis in cohabiting family members: a systematic review and meta-analysis
Source: J Oral Microbiol. 2019 Nov 7;12(1):1687398. doi: 10.1080/20002297.2019.1687398 (PMC6844440; doi:10.1080/20002297.2019.1687398)
Supplement: Supplemental Material [file ZJOM_A_1687398_SM2275.docx]

**Supplemental Table 1**. Risk of bias for non-randomized clinical trials, cohort studies, case-series based on the Newcastle-Ottawa Scale

| Study/Criteria | Selection | Comparability | Exposure/Outcome | Overall Score |
| --- | --- | --- | --- | --- |
| Petit et al.1993 | * | - | ** | 3/9 |
| Saarela et al. 1993a | * | - | ** | 3/9 |
| Van Steenbergen. 1993a | * | - | ** | 3/9 |
| Van Steenbergen. 1993b | * | - | ** | 3/9 |
| Petit et al.1994 | * | - | ** | 3/9 |
| Von Troil Linden. 1995 | ** | - | *** | 5/9 |
| Asikainen et al. 1996 | * | - | ** | 3/9 |
| Van der Welden. 1996 | ** | - | ** | 4/9 |
| Tuite McDonnell et al. 1997 | ** | - | ** | 4/9 |
| Von troil Linden. 1997 | ** | - | ** | 4/9 |
| Van Winkelhoff .1999 | ** | - | *** | 5/9 |
| Asano et al. 2003 | * | - | ** | 3/9 |
| Park et al. 2004 | * | - | ** | 3/9 |
| Umeda et al. 2004 | * | - | ** | 3/9 |
| Okada et al. 2004 | * | - | ** | 3/9 |
| Tamura et al. 2006 | * | - | ** | 3/9 |
| Rijnsburger et al. 2007 | * | - | ** | 3/9 |
| Van Winkelhoff. 2007 | ** | - | ** | 4/9 |
| Van Winkelhoff. 2008 | ** | - | ** | 4/9 |
| Kobayachi et al. 2008 | * | - | ** | 3/9 |
| Belcheva et al. 2012 | * | - | ** | 3/9 |
| Martelli et al 2012 | * | - | ** | 3/9 |
| Feng. 2015 | * | - | ** | 3/9 |
| Monteiro et al. 2014 | ** | - | *** | 5/9 |
| Monteiro et al. 2015 | ** | - | *** | 5/9 |
| Al Yahfoufi. 2017 | * | - | ** | 3/9 |
